# Simultaneous targeting therapy for lung metastasis and breast tumor by blocking the NF-κB signaling pathway using Celastrol-loaded micelles

**DOI:** 10.1080/10717544.2018.1425778

**Published:** 2018-01-22

**Authors:** Yue Zhao, Yanan Tan, Tingting Meng, Xuan Liu, Yun Zhu, Yun Hong, Xiqin Yang, Hong Yuan, Xuan Huang, Fuqiang Hu

**Affiliations:** a Ocean College, Zhejiang University, Zhoushan, China;; b College of Pharmaceutical Science, Zhejiang University, Hangzhou, China;; c The First Affiliated Hospital, College of Pharmaceutical Medicine, Zhejiang University, Hangzhou, China;; d Department of Pharmacy, School of Medicine Science, Jiaxing University, Zhejiang, China

**Keywords:** Simultaneous targeting, metastasis, tetraiodothyroacetic acid, breast tumor, drug delivery system

## Abstract

Metastasis is one of the major obstacles for successful therapy of breast tumor. To inhibit the metastasis and growth of breast tumor simultaneously, a Celastrol (Cela) loaded glucolipid-like conjugates (CSOSA/Cela) with αvβ3-ligand Tetraiodothyroacetic acid (TET) modification (TET-CSOSA/Cela) were established to block nuclear factor-kappa B (NF-κB) signaling pathway. The distribution of TET-CSOSA was remarkably increased in lung metastasis and primary tumor of 4T1 tumor-bearing mice by means of αvβ3 receptor-mediated interaction. The results demonstrated that TET-CSOSA/Cela significantly suppressed Bcl-2 activation of lung metastatic cells and reduced MMP-9 expression of 4T1 breast tumor cells by blocking NF-κB. The inhibitory rates of TET-CSOSA/Cela against lung metastasis and primary tumor were raised to 90.72 and 81.15%, compared to those of Celastrol (72.15 and 46.40%), respectively. All results demonstrated the αvβ3 receptor targeted TET-CSOSA/Cela micelles exhibited great potential in treating lung metastasis and primary tumor simultaneously via blocking NF-κB signaling pathway.

## Introduction

Most deaths from breast tumor are due to metastatic disease (O'Flanagan et al., 2017) and the five-year survival rate for advanced or metastasized breast tumor is only 26%. For many patients, metastasis has already occurred when tumor is detected (Klein, 2009; Ullah et al., 2017). If only treatment is given for primary tumor, metastasis may not be controlled, which may eventually result in treatment failure. Tumor cells invade over the barrier of degraded extra cellular matrix (ECM) via secreting matrix metalloproteinases (MMPs), which allows tumor cells to move forward (Valastyan & Weinberg, 2011; Zhu et al., 2012). Consequently tumor cells enter the bloodstream and a few lodge in distant organs such as the lungs (Woodhouse et al., 1997; Bonotto et al., 2014). Metastases acts as a biobarriers due to their smaller size, higher dispersion to organs, and lower vascularization than primary tumors, which make them less accessible to molecular drug (Nacev et al., 2011). However, metastatic lesions can upregulate specific cell-surface molecules and secreted factors that differ from the rest of its host organ (Ahmed & Douek, 2013). If the appropriate chemical specificity is selected, targeted micelles could provide a unique opportunity for therapeutic compounds to metastases and primary tumor.

As an important member of integrin family, the adhesion molecule αvβ3 has been widely investigated for tumor therapy and tumor angiogenesis (Hood & Cheresh, 2002; Rebbaa et al., 2008). The expression of integrin αvβ3 was upregulated on tumor vasculature and proliferating tumor endothelial cells when compared to resting endothelial cells and most normal tissues, as it played a key role in tumor migration, invasion, and metastasis (Schnell et al., 2008; Danhier et al., 2012). It has been shown that the metastatic site transitions from selectin-dependent tumor cell roll on the endothelium to firm attachment, which is mediated primarily by αvβ3 (Ahmed & Douek, 2013). Therefore integrin αvβ3 was considered as anideal target.

Active targeting therapy-mediated drug delivery system has been shown to reduce systemic toxicity and achieve targeted synergistic effects (Mo et al., 2014; Wang et al., 2014). Tetraiodothyroacetic acid (TET), a small molecule of a thyroid hormone analog, exhibits favorable binding affinity to integrin αvβ3 (Bergh et al., 2005; Mousa et al., 2008). TET conjugated liposomes can be used in diagnostic Positron-emission tomography (PET) imaging through binding to αvβ3 (Kang et al., 2013). However, there is little research on the application of TET to target lung metastases. TET could be used as a homing motif for directing drug delivery system to metastases and primary tumor.

Nuclear factor-kappa (NF-κB), a heterodimeric DNA-binding protein that consists of two major subunits, p50 and p65, has been found to play a crucial role in the growth progress and distant metastasis of human tumor (Perkins, 2007; Sethi et al., 2008; Meylan et al., 2009). Activated NF-κB can bind to DNA and lead to the expression of diverse genes that promote cell proliferation (Sakamoto & Maeda, 2010), regulate apoptosis, facilitate angiogenesis, and stimulate invasion and metastasis (Huang et al., 2000; Park et al., 2007; Wu et al., 2009). Thus, targeting and intervening this signaling pathway using drug delivery system could prevent tumor metastasis and consequently reduce mortality. Celastrol is a natural proteasome inhibitor that has been reported to show anti-metastasis and apoptosis effects in breast tumor models (Pang et al., 2010; Zheng et al., 2014; Kapoor, 2016). Studies have shown that Celastrol has a pharmacological effect of inhibiting NF-κB, which is involved in the regulation of genes expression that promote the growth and metastasis of tumor (Zhou et al., 2011; Sha et al., 2014), as anti-apoptotic proteins Bcl-2 (Sethi et al., 2013; Lu et al., 2015) and proteases MMP-9 (Kim et al., 2009; Mi et al., 2014). However, the poor aqueous solubility and low therapeutic index of Celastrol limited its clinical application (Tan et al., 2017). Therefore, it is necessary to improve the solubility and achieve the high concentration of Celastrol in the metastases and primary tumor by means of the active targeting drug delivery technique.

**Scheme 1.**
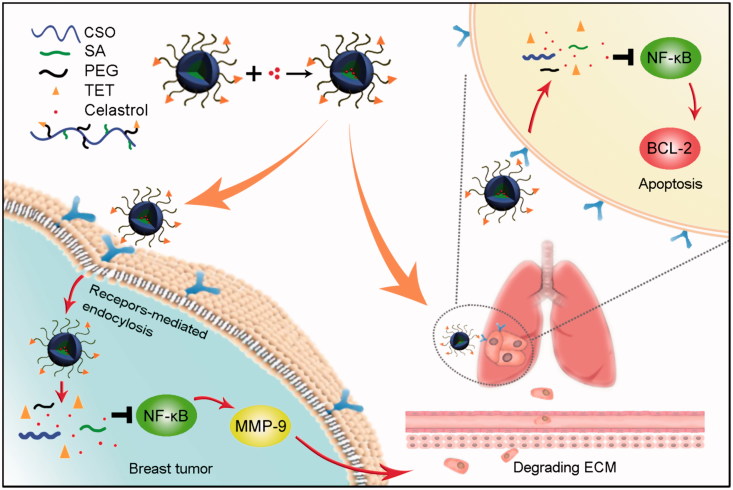
The schematic diagram of simultaneous targeting therapy for lung metastasis and breast tumor.

In order to treat lung metastasis and primary tumor successfully, in this article, a new αvβ3 targeted drug delivery system was designed and developed with encapsulating Celastrol. The drug delivery system enhanced accumulation in the metastasis nodes through αvβ3 receptor-mediated interaction. This study further explored the therapeutic effect, mechanism of drug delivery system, and elucidated the reasons for its role in the treatment of lung metastasis and primary tumor (Scheme 1).

## Materials and methods

### Materials

Chitosan oligosaccharide (CSO) of low molecular weight (M_w_ =20 kDa) was obtained by enzymatic degradation of chitosan (95% deacetylated, M_w_ = 450 kDa, supplied by Yuhuan Marine Biochemistry Co., Ltd, Zhejiang, China). Stearic acid (SA) was purchased from Shanghai Chemical Reagent Co., Ltd (Shanghai, China). NH_2_-PEG_2000_-NH_2_, fluorescein isothiocyanate (FITC) and 3-(4,5-dimethylthiazol-2-yl)-2,5-diphenyltetrazolium bromide (MTT) were obtained from Sigma-Aldrich Inc. (St Louis, MO). TET was from Wuhan FengYuan Technologies Inc (Wuhan, China). N,N′-Disuccinimidyl carbonate (DSC) was obtained from Bio Basic Inc. (Toronto, Canada). Ditertbutyldicarbonate ((Boc)2 O) and 1-ethyl-3-(3-dimethyl-aminopropyl) carbodiimide (EDC) were purchased from Shanghai Medpep Co, Ltd (Shanghai, China). Pyrene was purchased from Aldrich Chemical Co (Milwaukee, WI). Celastrol was supplied by Dalian Meilun Pharm Co., Ltd. (Dalian, China). 1,1′-Dioctadecyl-3,3,3′,3′-tetramethyl indotricarbocyanine iodide (DiR) was supplied by Life Technologies (Carlsbad, CA, USA). 1, 1′-dioctadecyl-3, 3, 3′, 3′-tetramethylindocarbocyanine perchlorate (Dil) was supplied by the Beyotime (Shanghai, China). Chitosanase was purchased from Dyadic International Inc. (Jupiter, Florida). Trypsin and Dulbecco’s modified Eagle’s medium were purchased from Gibco-BRL Life Technologies (Carlsbad, CA). Fetal bovine serum was purchased from Sijiqing Biology Engineering Materials Co, Ltd (Zhejiang, China). Other chemicals used were of analytical or chromatographic grade.

### Synthesis of TET modified CSOSA

CSOSA was prepared as described before (Du et al., 2012). Briefly, SA and EDC were dissolved in acetone and then added into ethanol-acetone mixed solvent (ethanol: acetone = 3:7, v/v). The mixture was stirred for 0.5 h at 60 °C. CSO (M_w_ = 20 kDa) was dissolved in hot water when the SA and EDC mixture was added dropwise. The reaction solution was stirred for another 4 h and then was dialyzed against DI water using a dialysis membrane (MWCO: 7 kDa, Spectrum Laboratories, Laguna Hills, CA) for 48 h. Then the product was collected by lyophilization following further wash with ethanol to remove products. The washed product was collected by lyophilization again.

To synthesize TET modified CSOSA, NH_2_-PEG_2000_-NH_2_ was used to connect the TET and CSOSA. 20 mg of TET, 15 mg of EDC and 10 mg of NHS were dissolved in DMSO and the mixture was stirred for 2 h at room temperature. Then, 50 mg of NH_2_-PEG_2000_-NH_2_ was dissolved in DMSO, the previous mixture was added dropwise and stirred for another 8 h. Consequently, 7 mg of DSC was added and stirred for 12 h. After that, 50 mg of CSOSA was dissolved in 10 mL of deionized (DI) water, the above mixture was added dropwise and stirred for another 24 h. The final reaction product was dialyzed against DI water for 72 h and then was collected by lyophilization.

### Preparation of CSOSA/Cela and TET-CSOSA/Cela micelles

Celastrol was used as the model drug which was prepared from a method used in a previous study (Liu et al., 2016). Briefly, 10 mg of polymers was dispersed in 5 mL of DI water and Celastrol was dissolved in DMSO of 5 mg/mL. Then, Celastrol/DMSO was gradually added into the polymers solution. After stirring for 2 h in dark, the mixture was dialyzed. Finally, the product was obtained by centrifuging at 8000 rpm for 10 min to get rid of un-encapsulated Celastrol. Then, the amount of encapsulated Celastrol in micelles was detected by an high performance liquid chromatography (HPLC) system. The mobile phase consisted of methanol-water containing 1% acetic acid (95:5, v/v) at a rate of 1 mL/min at 35 °C. An ultraviolet detector was set to 425 nm and linked to Chem-Station software for data analysis. The drug loading content (DL%) and drug encapsulation efficiency (EE%) were counted according to the Equations (1) and (2):

(1) $$\text{DL}\left(\%\right)\text{=}\frac{\text{W}\text{eight}\;\text{of}\;\text{encapsulated}\;\text{Celastrol}}{\text{Weight}\;\text{of}\;\text{Celastrol-Celastrol}\;\text{loaded}\;\text{micelles}}\text{×100\%}$$

(2) $$\text{EE}\left(\%\right)\text{=}\frac{\text{W}\text{eight}\;\text{of}\;\text{encapsulated}\;\text{Celastrol}}{\text{Weight}\;\text{of}\;\text{Celastrol}\;\text{in}\;\text{feed}}\text{×100\%}$$

### Characteristics of TET-CSOSA and Celastrol-loaded micelles

A ^1 ^H NMR spectrometer (AC-80, Bruker Biospin, Germany) was used to obtain ^1 ^H NMR spectra of chemicals. Celastrol was dissolved in deuterated DMSO, while CSOSA, NH_2_-PEG_2000_-NH_2_, and TET-CSOSA polymers were dissolved in heavy water (D_2_O) at the concentration of 10 mg/mL. The degree of amino substitution (SD%) of CSO on micelles was detected by 2,4,6-trinitrobenzenesulfonic acid (TNBS) test. A Zetasizer (3000HS, Malvern Instruments Ltd, UK) was used to examine the hydrodynamic diameters and zeta potentials of blank and Celastrol-loaded micelles in DI water at the concentration of 1 mg/mL. A transmission electron microscope (TEM;JEOLJEM-1230, Japan) was used to observe the morphological characters of Celastrol-loaded micelles. The samples were placed on copper grids with films and stained with 2% (w/v) phosphotungstic acid for TEM viewing. Pyrene was used as a probe to determine the critical micelle concentration (CMC) of CSOSAand TET-CSOSA by fluorescence measurement. The intensity ratio of the first peak (I_1_, 374 nm) to the third peak (I_3_, 384 nm) and the polymer concentration were analyzed to calculate CMC.

### *In vitro* drug release

*In vitro* drug release profiles were conducted by dialysis diffusion method. Phosphate buffered saline (PBS, pH 7.4) was used as the dissolution medium for *in vitro* drug release determination. Each dialysis membrane bag (MWCO: 3.5 kDa) containing 1 mL Celastrol, CSOSA/Cela, or TET-CSOSA/Cela solution was placed in a tube containing 20 mL PBS (Fit the slot condition). The tubes were put in an incubator shaker (HZ-8812 S-B, Hualida Laboratory Equipment Company, Tai Cang, China), which kept shaking horizontally at 75 rpm at 37 °C. At point-in-time, the dissolution medium out of the dialysis membrane was poured out and replaced by fresh medium. Drug concentration was then determined by using a HPLC system. All the tests were repeated thrice.

### Cell culture

4T1 and human breast cell line MDA-MB-231 cells were cultured at 37 °C in DMEM medium supplemented with 10% fetal bovine serum in a humidified atmosphere containing 5% CO_2_. Primary human umbilical vascular endothelial cells (HUVECs) were cultured in Roswell Park Memorial Institute (RPMI) *1640* Medium supplemented with 10% fetal bovine serum in a humidified atmosphere containing 5% CO_2_.

### Cellular uptake

CSOSA and TET-CSOSA micelles were labeled with FITC (FITC: micelles = 2:1, mol/mol). 4T1 and MDA-MB-231 cells were cultured in the 24-well plate and incubated for 24 h for attachment. Subsequently, FITC labeled CSOSA and TET-CSOSA micelles (20 μg/mL) were injected and the cells were further incubated for 2,4 and 8 h, respectively. Hochest 33342 was used to stain the cell nucleus. After washing the cells with PBS, the cell samples were observed by confocal laser scanning microscopy (CLSM;Carl Zeiss LSM 510, Germany). The rate of cellular uptake on CSOSA and TET-CSOSA was evaluated by using a BD FACSCalibur Flow Cytometer (BD Biosciences, San Jose, CA). Briefly, CSOSA and TET-CSOSA were labeled with FITC and 1 × 10^6^ cells were collected as described before. Flow cytometry results were obtained on the same machine at the same settings on the same day.

To study the competitive inhibition, cells were incubated with the free TET 80,40,20, and 0 μM, respectively, for 2 h before adding FITC labeled TET-CSOSA micelles and incubated for 24 h as described before. Flow cytometry results were also obtained as described before.

### Cytotoxicity and apoptosis *in vitro*

The cytotoxicity of drug-loaded micelles against 4T1 and MDA-MB-231 cells was evaluated with MTT assay. In summary, 5 × 10^3^ cells/well were seeded in a 96-well plate and maintained for 12 h at 37 °C. Then, a series of concentrations of micelles were added and cultured for additional 48 h. Then, 20 μL of MTT at a concentration of 5 mg/mL was added into the cells which were kept for additional 4 h incubation. The medium in each well was replaced with 200 μL of DMSO to dissolve the formazan crystals. Finally, the absorbance of samples at 570 nm was determined using an automatic reader (BioRad, Model 680, Hercules, CA). For the Annexin V apoptosis assay, 4T1 and MDA-MB-231 cells were cultured with 1 µg/mL Celastrol, CSOSA/Cela, and TET-CSOSA/Cela for 6 h, then were subsequently incubated with Annexin-V antibodies (BD Biosciences) for the detection of apoptosis. Flow cytometry results were obtained as described before.

### Uptake of TET-CSOSA by multicellular spheroids

4T1 multicellular spheroids were produced by the liquid overlay method. Briefly, 4T1 cells (10 µL per well containing 5 × 10^3^ cells) were transferred into flat-bottomed 96-well plates pre-coated with 2% agarose. Cells were incubated for approximately five days, as described above for monolayer cells. The culture medium was partially (0.1 mL) replaced with fresh medium every other day. 4T1 spheroids with diameters of approximately 300 − 400 μm were harvested and incubated with CSOSA/Dil and TET-CSOSA/Dil for 24 h. The medium was then removed and the spheroids were washed with ice-cold PBS. 4T1 cell spheroids were imaged in every 9 μm sections of tissue from the top to the middle using CLSM.

### TET-CSOSA/Cela against multicellular spheroids

4T1 spheroids with diameters of 200 − 300 μm were divided into four groups (*n* = 5 per group). The selected spheroids were treated with fresh medium, Celastrol, CSOSA/Cela, and TET-CSOSA/Cela (1 μg/mL). The spheroids were further allowed incubated at 37 °C for seven days. As an indication of multicellular spheroid proliferation, the diameter of the spheroids was measured every day using an optical microscope (Olympus, Japan).

### Anti-metastasis effect *in vitro*

The wound healing, transwell migration, and invasion assays were utilized to evaluate *in vitro* anti-metastasis effect of micelles on two metastatic breast tumor cells. For wound healing assay, 4T1 and MDA-MB-231 cells were cultured for 24 h in 12-well culture plates at 2 × 10^5^ cells per well. The grown confluent cell monolayers were scratched by a 20 µL pipette tip and rinsed twice with PBS. Then, the cells were respectively treated for 24 h with fresh medium (as control), Celastrol, Celastrol loaded CSOSA, and TET-CSOSA at a concentration of 0.5 µg/mL under 5% CO^2^ at 37 °C for 12 h. After another two rinses with PBS, the reparation of scratched area of each sample was viewed by an optical microscope. For cell migration assay, 4T1 or MDA-MB-231 cells were harvested and suspended at a density of 1 × 10^5^ cells/mL in FBS-free culture medium and 100 mL of cell suspensions were seeded on the upper surface of transwell chamber (Corning Incorporated, Corning, NY). The outside chamber was filled with 600 µL of culture medium with 10% FBS which was used as a chemoattractant. Then, the cells were respectively treated for 6 h with serum-free medium (as control), Celastrol, Celastrol loaded CSOSA, and TET-CSOSA at a concentration of 0.5 µg/mL. After that, cells on the upper surface of membrane (non-migration cells) were scrubbed off with humid cotton buds, while cells on the bottom surface (migration cells) were immobilized with cold 70% ethanol, stained with 0.1% crystal violet for 30 min, and viewed by an optical microscope The cell invasion assay was carried out with 1 × 10^5^ cells/well similar to cell migration assay as described above in the transwell chambers coated with Matrigel layer.

### Capillary-like tube formation assay

Tube formation was assessed as described previously (Pang et al., 2009). Briefly, HUVECs were seeded onto the Matrigel layer in 96-well plates at a density of 5 × 10^4^ cells, at the same time PBS, Celastrol, CSOSA/Cela, and TET-CSOSA/Cela (0.5 µg/mL) was also added, after 6 h, tubular structure of endothelial cells was photographed using an inverted microscope (Olympus, Japan). Three independent experiments were performed.

### Western blot analysis

4T1 cells seeded in 24-well plates were treated with Saline, Celastrol, CSOSA/Cela, and TET-CSOSA/Cela at the concentration of 1 μg/mL for 24 h. Cells were then harvested, washed twice with cold PBS and lysed. Equal amounts of proteins were separated on a 10% sodium dodecyl sulfate (SDS) -polyacrylamide gel and transferred onto nitrocellulose membranes (Millipore, Billerica, MA). The proteins were identified by incubation with primary antibodies against NF-κB, MMP-9, Bcl-2, and β-actin (Abcam, Cambridge, UK), followed by incubation with horseradish-peroxidase conjugated secondary antibody (Beyotime Biotechnology, Jiangsu, China).

### *In vivo* bio-distribution

All animal procedures were performed according to national regulations and was approved by Ethics Committee of the Institution on Laboratory Animal Care in Zhejiang University. Tumors on the right mammary gland of female BALB/c mice were initiated by injection of 1 × 10^5^ 4T1 cells. Tumors were allowed to grow to a volume of around 100 mm^3^. Female mice bearing metastatic 4T1 breast tumor were randomly assigned to two groups and injected through the tail vein with DiR loaded CSOSA and TET-CSOSA micelles (5 mg/kg of DiR), respectively. After 48 h, the mice were sacrificed, which was followed by collection of heart, liver, spleen, lung, kidney, and tumor. The fluorescence images were obtained by using a Maestro *in vivo* Imaging System (CRI Inc., Woburn, MA).

For the lung metastasis imaging, primary 4T1 tumor-bearing mouse model was established as before. When tumor sizes reached approximately 100 mm^3^, female mice were randomly assigned to two groups and DiR loaded CSOSA and TET-CSOSA micelles (5 mg/kg of DiR) were injected through the tail vein as before. The above operation was repeated in 10 and 21 days respectively. After 48 h, the mice were sacrificed and the lung tissues were dissected for *ex vivo* fluorescent imaging.

### Inhibition of growth and metastasis of breast tumor *in vivo*

For *in vivo* antitumor experiment, female BALB/c mice were randomly assigned to experimental and control groups (*n* = 5). The primary of murine carcinoma were developed by implanting 1 × 10^5^ 4T1 cells to the right mammary gland. When the tumors volume reached around 100 mm^3^, animals were injected through the tail vein with saline, Celastrol, CSOSA/Cela, and TET-CSOSA/Cela 2 mg/kg every two days for five times. Animal weight and tumor volume were measured every three days for three weeks. Individual tumor volume was calculated. After three weeks, animals were sacrificed for humane reasons; the tumor and lung tissue was immediately excised, imaged and were frozen for hematoxylin and eosin (H&E) staining.

### Statistical analysis

All the data were expressed as the mean values ± standard deviation (SD). Statistically significant differences between groups were determined with Student’s *t*-test. *p* values < .05 were regarded statistically significant.

## Results and discussion

### Synthesis and characteristics of TET-CSOSA

**Figure 1.**
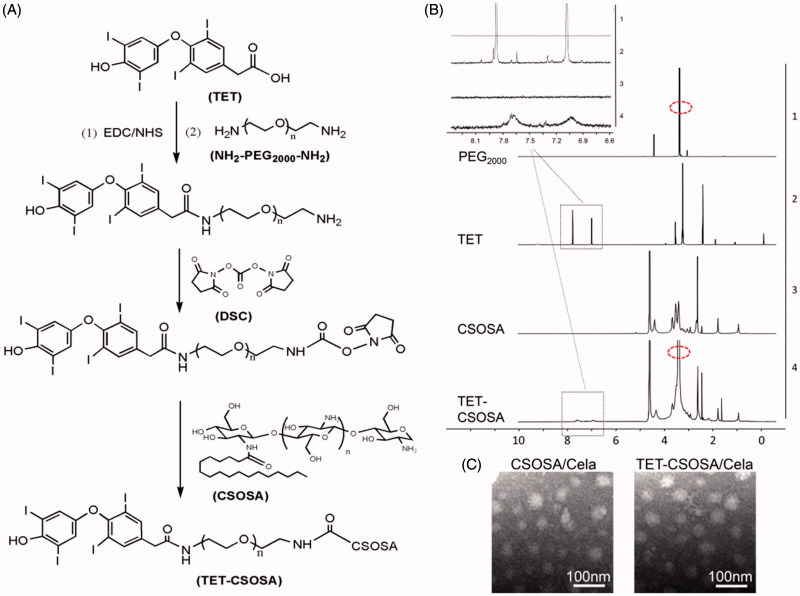
Synthesis route, ^1 ^H NMR spectra, and characterization of TET-CSOSA micelles. (A) Synthetic route of TET-CSOSA. (B) ^1 ^H NMR spectra of NH_2_-PEG_2000_-NH_2_, TET, CSOSA, and TET-CSOSA. The representative peaks were pointed out and magnified (upper). (C) TEM images of the CSOSA/Cela and TET-CSOSA/Cela micelles.

Briefly, the CSOSA conjugate was obtained by a coupling reaction between amino groups of CSO and carboxyl groups of SA in the presence of EDC. As shown in Figure 1(A), TET-PEG was prepared by the chemical reaction between TET and NH_2_-PEG_2000_-NH_2_ in the presence of EDC and NHS. DSC was utilized as a versatile reagent for active ester synthesis between residual amino group of TET-PEG and those remaining on CSOSA via amide reaction. Finally the obtained TET-CSOSA was white soluble powder after lyophilization.

The chemical structures of TET-CSOSA were confirmed by ^1 ^H NMR (Figure 1(B)). The peaks at about 1.90, 2.28 and 3.77 ppm were attributed to –CH_3_, –NH_2_, and –COOH on CSOSA, respectively. The peak at about 3.54 ppm (circled with a red ellipse) was attributed to –CH_2_CH_2_O– of NH_2_-PEG_2000_-NH_2_. The tiny but indelible waves near the peaks at 7.03 and 7.69 ppm belonged to the benzene ring of TET. These results indicated that the TET was conjugated to CSOSA.

The synthesized CSOSA and TET-CSOSA could be micellization and self-assembled in aqueous solution. The average sizes of CSOSA and TET-CSOSA were 137.0 ± 14.7 and 104.8 ± 7.3 nm and zeta potentials were 35.9 ± 1.2 (CSOSA) and 33.3 ± 0.7 mV (TET-CSOSA), respectively. TET-CSOSA obtained was smaller in size than CSOSA due to the modification of hydrophobic TET. The degrees of amino-substitution (SD%) of CSOSA and TET-CSOSA were measured as 6.55 and 8.49%, respectively, it was calculated that CSOSA was modified with 1.94% of TET on TET-CSOSA. The relationship between the I_1_/I_3_ ratio and the logarithmic concentration (log C) of CSOSA and TET-CSOSA are presented in Supplementary Figure S1(A) and CMC of CSOSA and TET-CSOSA were 69.2 and 61.6 μg/mL, respectively.

### Preparation and characterization of Celastrol-loaded micelles

The particle size and zeta potential of Celastrol-loaded micelles are presented in Supplementary Table S1. The average size of CSOSA/Cela and TET-CSOSA/Cela were 84.6 ± 1.5 and 82.5 ± 3.6 nm, respectively. Since Celastrol brought stronger cohesive force inside the hydrophobic core of micelles, CSOSA/Cela and TET-CSOSA/Cela represented smaller size than CSOSA and TET-CSOSA. Zeta potential of CSOSA/Cela and TET-CSOSA/Cela were positive in DI water. Celastrol was encapsulated in CSOSA and TET-CSOSA micelles with an efficiency of 81.1 ± 7.9 and 73.3 ± 3.9%. *In vitro* Celastrol release profile is shown in Supplementary FigureS1(B). There was no obvious difference between CSOSA/Cela and TET-CSOSA/Cela, which indicated that TET and PEG_2000_ rarely influence drug release. The cumulative drug release curve showed a typical biphasic pattern and about 80% drug was released in 60 h.

### Cellular internalization

**Figure 2.**
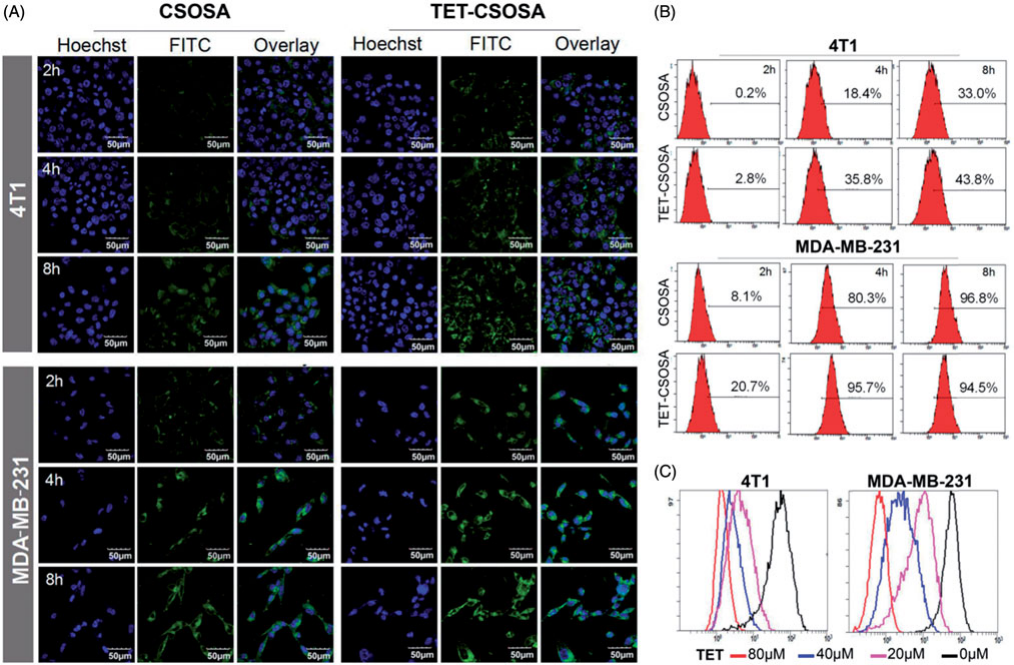
Fluorescence analysis of the cellular uptake and competition assay of TET- CSOSA in 4T1 and MDA-MB-231 cells. (A) Confocal laser scanning microscopy images of the cellular uptake of FITC labeled CSOSA and TET-CSOSA for 2, 4, and 8 h. All scale bars represent 50 μm. (B) Quantitative evaluation of the cellular uptake based on flow cytometry. (C) *In vitro* competition assay for detecting the targeting of TET-CSOSA to αvβ3 in 4T1 and MDA-MB-231 cells. Cells were preincubated with 0μ, 20, 40, and 80 μΜ of TET, respectively. Fluorescence signals were observed by flow cytometry.

*In vitro* cellular uptake of FITC labeled micelles on 4T1 and MDA-MB-231 cells was visualized by CLSM at 2, 4, and 8 h, respectively. Figure 2(A,B) showed that FITC labeled TET-CSOSA had remarkably higher cell uptake with positive rate of 35.8% compared to unmodified FITC labeled CSOSA which had a rate of 18.4% in 4T1 at 4 h and the cell uptake in MDA-MB-231 cells showed similar trend. The cellular uptake of micelles in both cell lines was time dependent. These results made it clear that TET-CSOSA micelles displayed a higher binding affinity to 4T1 and MDA-MB-231.

Furthermore, we conducted the competitive inhibition studies to verify whether TET-CSOSA micelles were internalized via αvβ3 receptor-mediated pathway. After preincubation with 0,20,40 and 80 μM, respectively of free TET, cellular uptake rate of FITC labeled TET-CSOSA on 4T1 and MDA-MB-231 cells were measured by flow cytometry (Figure 2(C)). It showed that the uptake rate of TET-CSOSA decreased with the increase of TET preincubation concentration in both cell lines, which further confirmed the results that are shown in Figure 2(A). The above results established that TET-CSOSA was internalized via αvβ3 receptor-mediated pathway, which could be utilized as a potential active targeting vector.

### Cytotoxicity and apoptosis of micelles *in vitro*

To examine the cytotoxicity of Celastrol, CSOSA/Cela, and TET-CSOSA/Cela on 4T1 and MDA-MB-231 cells, the MTT assay was performed. Supplementary Figure S2(A,B) showed that TET-CSOSA/Cela micelles exhibited a stronger inhibition effect than Celastrol with an IC_50_ value of 0.54 ± 0.01 μg/mL in 4T1 cells and 1.11 μg/mL in MDA-MB-231 cells, compared with Cela 0.88 ± 0.02 and 1.53 ± 0.03 μg/mL, which may be due to the quick cellular uptake of TET-CSOSA/Cela with high concentration gradient. IC_50_ values of blank CSOSA and TET-CSOSA micelles against both tumor cells were all above 500 μg/mL (Supplementary Figure S2(C,D)), which implied that CSOSA and TET-CSOSA micelles had relatively low cytotoxicity.

To further understand the underlying mechanisms of the enhanced cytotoxicity observed with TET-CSOSA/Cela, we examined their effects on apoptosis. Annexin V staining for phosphatidylserine redistribution to the outer plasma membrane was employed to measure apoptosis in 4T1 and MDA-MB-231 cells, which were exposed to Celastrol, CSOSA/Cela, and TET-CSOSA/Cela for 6 h. As shown in Figure 4(E), 48.3% of the cells were found apoptotic after being treated with TET-CSOSA/Cela, in contrast to CSOSA/Cela (30.1%) and Celastrol (23.2%). Accumulating evidence has implicated that NF-κB could mediate tumor cells proliferation and apoptosis (Aggarwal & Das, 2016). Anti-apoptotic activity of NF-κB involves the inhibition of Tumor necrosis factor(TNF) through induction of a variety of anti-apoptotic proteins including Bcl-2, cIAPs, Bcl-XL, and Gadd45b (Sethi et al., 2013). In this study, the effect of TET-CSOSA/Cela on Bcl-2 protein was investigated by western blot analysis. Bcl-2 expression in the TET-CSOSA/Cela group was seen to be the least. Together with apoptosis and cytotoxicity results, it is highlighted that TET-CSOSA/Cela had a potential to be used in tumor therapy.

### Anti-metastasis assay of Celastrol loaded micelles *in vitro*

**Figure 3.**
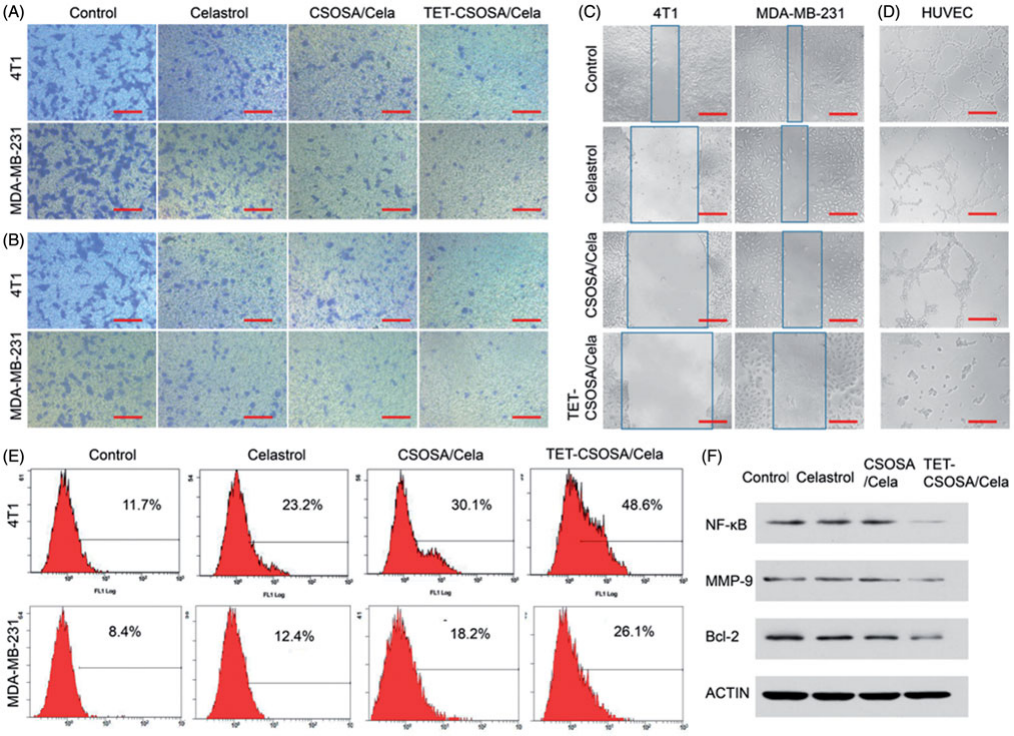
*In vitro* apoptosis and anti-metastasis effects of different formulations against 4T1 and MDA-MB-231 cells. Optical images of cells migration, (A) invasion, (B) and wound edge (C) after incubation for 12 h with 0.5 µg/mL of Celastrol, CSOSA/Cela, and TET-CSOSA/Cela. (D) Inhibition for the tube formation in HUVECs cells (bar =200 µm). (E) Apoptosis of Celastrol and Celastrol loaded micelles in 4T1 and MDA-MB-231 cells tested by Annexin V and analyzed by flow cytometry respectively. (F) The expression levels of NF-κB, MMP-9, and Bcl-2 in 4T1 cells were detected by Western blot.

Cell invasion and migration are necessary steps involved in tumor progression to metastasis (Matsumoto et al., 2016). We performed transwell migration (Figure 3(A)), invasion assay (Figure 3(B)), and monolayer scratch assay (Figure 3(C)) to assess the anti-metastatic ability of Celastrol and Celastrol loaded micelles, analysis results are presented in Supplementary Figure S2. Control groups demonstrated that both 4T1 and MDA-MB-231 possessed superior metastatic characters. Compared to Celastrol and CSOSA/Cela, TET-CSOSA/Cela exerted much more potency to inhibit cellular motilities. As is shown in Figure 3(B) and Supplementary Figure S3(B), Celastrol slightly decreased cell invasion in both 4T1 and MDA-MB-231 cells, there was a 1.5-fold decrease of CSOSA/Cela and a 2.9-fold decrease of TET-CSOSA/Cela compared to Celastrol in 4T1 cells, which may have resulted in the increased ECM degrade by TET-CSOSA/Cela which may have tumor cell invasion to the bottom chamber.

To explore the underlying molecular mechanism of inhibited invasion, we examined the regulation of NF-κB/MMP-9 in tumor cells after treating it with TET-CSOSA/Cela. In Figure 3(F), Western blot analysis showed that both Celastrol and CSOSA/Cela diminished the expression of NF-κB/MMP-9 slightly, while TET-CSOSA/Cela significantly decreased them by means of more uptake in 4T1 cells. Studies have been reported that the human MMP-9 promoter contains cis-acting regulatory elements and the transcription factor binding sites including NF-κB (-600 bp) and AP-1 (-533 bp, -79 bp) binding sites, which participate in regulation of the MMP-9 gene transcription. The released NF-κB translocates into the nucleus and binds to the promoter region of MMP-9, leading to gene expression (Yang et al., 2015). These results indicated that TET-CSOSA/Cela significantly suppressed the breast tumor cell invasion via down regulating MMP-9 expression controlled by NF-κB.

To further determine the effect of TET-CSOSA/Cela on angiogenesis, we examined how Celastrol regulated capillary tubule formation of HUVECs. When HUVECs were seeded on the two-dimensional Matrigel, robust tubular like structures were formed. However, TET-CSOSA/Cela abolished tubule formation of HUVECs compared to the groups of Control, Celastrol, and CSOSA/Cela (Figure 3(D) and Supplementary Figure S3(D)), suggesting the potential effect of TET-CSOSA/Cela on angiogenesis. Angiogenesis is one of the key processes that mediate metastasis. Studies have shown that Celastrol could mediate growth and metastasis of tumor through suppression of angiogenesis (Huang et al., 2011). Once angiogenesis was blocked by TET-CSOSA/Cela, tumor growth and metastasis were substantially suppressed.

### Inhibition effect of multicellular spheroids

Multicellular tumor spheroids, one of the most widely used three-dimensional (3 D) culture systems, have been shown to have many advantages over two-dimensional (2 D) culture systems for tumor research (Ducommun & Lobjois, 2014). TET-CSOSA/Dil had remarkably higher cell uptake relative to CSOSA/Dil in every section of tissue on 4T1 (Supplementary Figure S4(A)). We further investigated inhibition effect of TET-CSOSA/Cela on 4T1 cells spheroids *in vitro*. 4T1 cells spheroids were exposed to 1 μg/mL of Celastrol, CSOSA/Cela, and TET-CSOSA/Cela on day 1. The spheroids in the control group grew continuously throughout the entire experimental period. However, the growth of the spheroids in all Celastrol-loaded micelles groups ware suppressed (Supplementary Figure S4(B)). On day 5, the growth inhibition rate of Celastrol, CSOSA/Cela, and TET-CSOSA/Cela treated groups were 21.6, 35.2 and 46.5%, respectively, compared to the spheroid volume of the control group (Supplementary Figure S4(C)). The spheroid volume of TET-CSOSA/Cela treated group was markedly smaller (35.3%) on day 5 in comparision to day 1, compared to CSOSA/Cela treated group (19.6%), while volume slightly increased by Celastrol treated group, suggesting that the cells in the outer layers of the spheroids were efficiently killed due to the apoptosis effect of TET-CSOSA/Cela (Supplementary Figure S4(D)). The high cellular uptake of TET-CSOSA/Cela via αvβ3 receptor-mediated pathway may largely explain why it demonstrated the best inhibitory effect against the tumor spheroids.

### Distribution of micelles in breast tumor bearing mice

**Figure 4.**
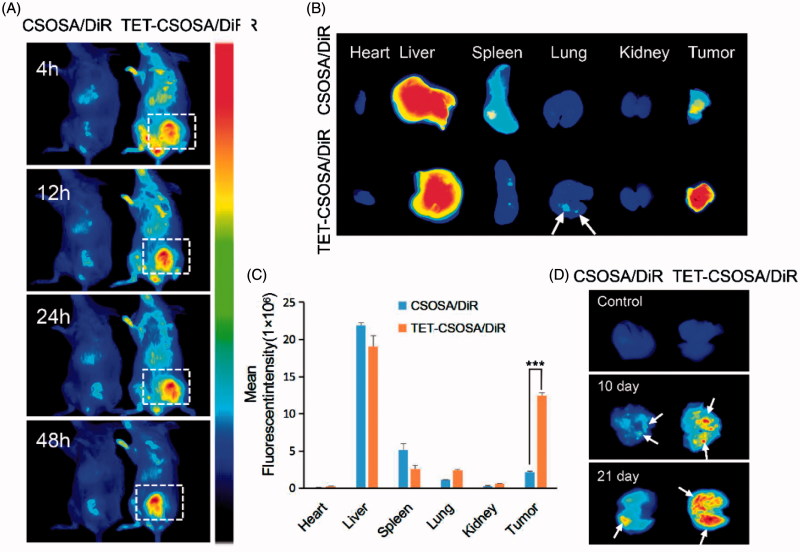
*In vivo* simultaneous distribution in 4T1 metastasis model. *In vivo* targeting of CSOSA/DiR and TET-CSOSA/DiR to breast 4T1 tumor (A), dissected tumors and organs, (B) and quantification of the mean fluorescent intensity (C) after 48 h of injection (*n* = 3). (D) Fluorescence imaging of the dissected lungs after CSOSA/DiR and TET-CSOSA/DiR treatment in the 4T1 lung metastasis tumor bearing mice. ***p < .001.

The distribution of CSOSA/DiR and TET-CSOSA/DiR in major organs was determined in a well-established 4T1 pulmonary metastasis tumor model, which was generated by subcutaneous injection of 4T1 cells into the mammary fat pad of female BALB/c mice. On the day 10 the size of the breast tumor was about 100 mm^3^. CSOSA/DiR and TET-CSOSA/DiR was administered intravenously, both micelles were seen to be swiftly distributed all over the body with noticeable signals at tumor site since 4 h post-injection (Figure 4(A)). More intense and lasting fluorescent signals (5.69 times) were observed in the tumor tissue of TET-CSOSA/DiR group compared with CSOSA/DiR group, which suggesting highly specific tumor accumulation of TET-CSOSA/DiR (Figures 4(B) and 6(C)). More detectable fluorescence was found to accumulate in the lungs metastasis compared to CSOSA/DiR as the white arrow of Figure 4(B), which meant simultaneous targeting of TET-CSOSA/DiR to primary tumor and metastasis. Much stronger signals were observed to spread all over the lung tissues for TET-CSOSA/DiR on day 10 and 21, especially in tumor nodules (white arrow marked), which indicated superior metastasis targeting capability of TET-CSOSA/DiR over CSOSA/DiR. Moreover, there were rather weak fluorescent signals in the normal lungs for both micelles. It deduced that TET-CSOSA/DiR were prone to be distributed in the tumor metastasized lungs rather than the corresponding normal lungs, due to the capability to distinguish diseased from normal tissues via αvβ3 receptor-mediated interaction. These results demonstrated that αvβ3 active targeting moiety of TET facilitated primary tumor and metastasis tissue-selective recognition.

### Inhibition of primary tumor and metastasis *in vivo*

**Figure 5.**
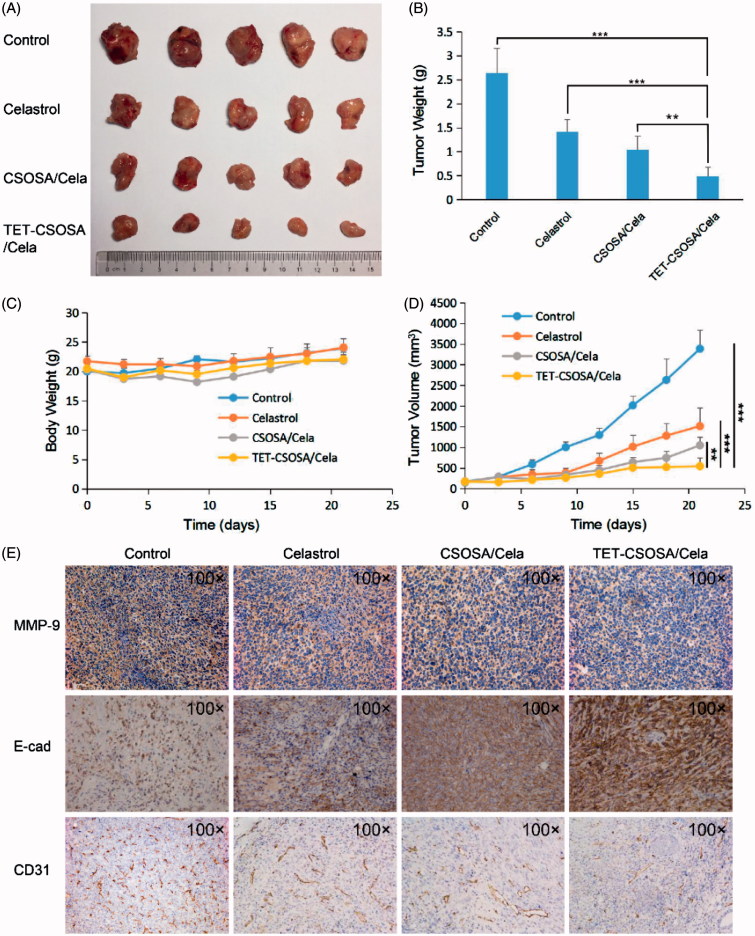
Therapeutic effects on 4T1 breast primary tumor bearing mice after intravenous administration with different formulations at a drug dose of 2 mg/kg. Images of the excised tumors at the end of the tests: dissected tumors weight (A), tumor weight-time graph (B), body weight-time graph (C), and tumor volume-time graph (D) during the treatment (*n* = 5). (E) Histopathological examination of E-cad, CD31, and MMP-9 for breast tumor separated from saline, Celastrol, CSOSA/Cela, and TET-CSOSA/Cela mice at the end of experiment. **p < .01;***p < .001.

The capacity of TET-CSOSA/Cela to inhibit the growth and metastasis of tumor *in vivo* was evaluated in mice bearing 4T1 tumor. When tumor sizes reached approximately 100 mm^3^, the lungs were taken out and few metastasis nodules were found as shown in Supplementary Figure S5. Then mice were randomly divided into four groups and treated with saline, a drug dose of 2 mg/kg with Celastrol, CSOSA/Cela, and TET-CSOSA/Cela, respectively. Mice treated with saline showed rapid tumor growth, on the contrary, mice injected with TET-CSOSA/Cela resulted in considerable inhibition of tumor growth over a three-week time period. While the groups injected with Celastrol and CSOSA/Cela presented a certain degree of tumor development suppression (Figure 5(D)). At the end of the experiment, the tumor inhibition rate of Celastrol, CSOSA/Cela, and TET-CSOSA/Cela group was 46.40, 60.56 and 81.15% compared with saline group, respectively (Figure 5(A,B)). H&E images in Supplementary Figure S6 showed the pathological characters of tumor tissue in four groups. After being treated TET-CSOSA/Cela micelles, the tumor tissue was looser, compared with other three groups. Consistent with the bio-distribution results, it could be attributed to the enhanced tumor-targeting efficiency of TET-CSOSA/Cela by means of integrin αvβ3 targeting. As shown in Figure 5(C), each treated group displayed an increase in body weight to a certain extent, which was similar to the saline treated group. This indicated that TET-CSOSA/Cela micelles had little systemic toxicity and good biocompatibility. Slices of major organs, like heart, liver, spleen, lung, and kidney (Supplementary Figure S6), showed a great deal of metastasis in lung as well metastasis spots in liver (marked by yellow lines) for both control and Celastrol groups. Other organs showed no noticeable abnormalities or lesions compared to those from saline-treated mice, indicating the lack of appreciable organ damage and further suggesting the low toxicity of the Cela-loaded micelles.

**Figure 6.**
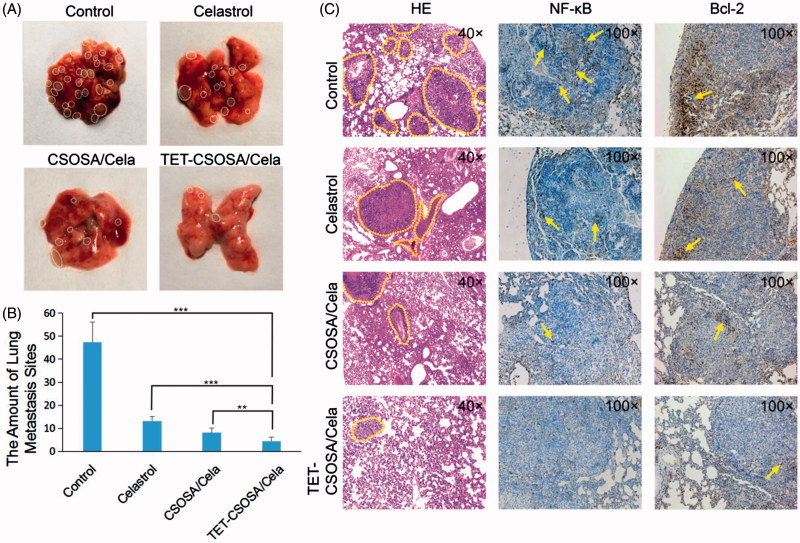
*In vivo* anti-metastasis experiment. (A) The photograph of metastasis sites in the lungs. (B) The amount of breast lung metastasis sites in 21 days (*n* = 5, mean ± SD). (C) Photos of the statistical analysis of metastasis sites on the lungs and histopathologic examination of NF-κB and Bcl-2 in mice lung metastasis at the end of treatment. **p < .01; ***p < .001.

As shown in Figures 6(A,B), the lungs from each group were taken out to count the lung metastasis nodules at the end of experiment. Compared with saline group, the amount of pulmonary metastatic nodules in mice treated with Celastrol, CSOSA/Cela, and TET-CSOSA/Cela decreased by 72.1, 82.7 and 90.7%, respectively. Metastasis inhibitory rate of TET-CSOSA/Cela was significantly higher than those of other groups, taking advantage of the higher distribution in lung tissues. In addition, the histological H&E staining results of lung sections further indicated that the suppression effect of TET-CSOSA/Cela on the formation of micro-metastatic foci in lungs was much better than those of other groups (Figure 6(C)). Simultaneous integrin αvβ3 targeting of TET-CSOSA/Cela showed more advantages for lung metastasis and breast tumor therapy when compared to CSOSA/Cela.

### Mechanisms of metastasis inhibition

To further investigate whether the anti-invasion efficiency of TET-CSOSA/Cela micelles was due to the inhibition of MMP-9 *in vivo*, we explored the expression of MMP-9 using immunohistochemistry. TET-CSOSA/Cela treatments had a markedly inhibitory effect on MMP-9, while CSOSA/Cela and Celastrol diminished slightly the expression of MMP-9, and similar results was obtained from MMP-9 Western blot analysis (Figure 3(F)). We next explored the expression of E-cadherin and CD31 using immunohistochemistry. As the most important marker for EMT, E-cadherin has been reported to be responsible for the intercellular connections and polarity (Heerboth et al., 2015). As expected, the expression of E-cadherin was significantly upregulated in TET-CSOSA/Cela treated group compared with those of other groups (Figure 5(E)). To investigate whether TET-CSOSA/Cela inhibited tumor angiogenesis, we used a blood vessel staining kit to stain solid tumor sections in the primary mouse model. We found that the number of blood vessels in TET-CSOSA/Cela group was the lowest when compared to those of other groups (Figure 5(E)). Combined with the regulation of E-cadherin and MMP-9, these results indicated that TET-CSOSA/Cela significantly inhibited tumor invasion, thereby exerting anti-metastatic effects *in vivo*.

Furthermore, to elucidate the molecular mechanism underlying the effect of TET-CSOSA/Cela against 4T1 lung metastasis, we next explored the expression of NF-κB and Bcl-2 in lungs using immunohistochemistry (Figure 6(C)). There was decreased NF-κB and Bcl-2 expression in TET-CSOSA/Cela treated group, highlighting the involvement of NF-κB suppression in the lung metastasis-growth inhibitory effects. The results were in agreement with the *in vitro* Western blot analysis data(Figure 3(F)). NF-κB signaling pathway promote the growth and metastasis of tumor by regulating positively the expression of genes, such as anti-apoptotic proteins Bcl-2, proteases that degrade the extracellular matrix (MMP-9), angiogenic factors VEGF, and E-cadherin, etc. (Sethi et al., 2013; Mi et al., 2014; Lu et al., 2015). The expression of these genes was significantly decreased by TET-CSOSA/Cela through blocking NF-κB activity, which resulted in significant inhibition of cell proliferation and invasion, thereby exhibiting an excellent anti-metastatic effect *in vivo*.

## Conclusion

In this study, we exploited a delivery system for improved antitumor metastasis therapy, which not only targeted breast tumor but also lung metastasis by means of αvβ3 receptor-mediated interaction. The results of 4T1 metastasis inhibition showed that TET-CSOSA/Cela could suppress breast tumor invasion and lung metastasis growth through inhibition of NF-κB signaling pathway. Overall, this simultaneous targeting delivery strategy could open a new avenue for treating breast metastasis.

## Supplementary Material

IDRD_Hu_et_al_Supplemental_Content.docx

## References

[CIT0001] Aggarwal S, Das SN. (2016). Garcinol inhibits tumour cell proliferation, angiogenesis, cell cycle progression and induces apoptosis via NF-kappa B inhibition in oral cancer. Tumor Biol 37:7175–84. 10.1007/s13277-015-4583-8 26662963

[CIT0002] Ahmed M, Douek M. (2013). The role of magnetic nanoparticles in the localization and treatment of breast cancer. Biomed Res Int 2013:281–30. 10.1155/2013/281230 PMC3722907 23936784

[CIT0003] Bergh JJ, Lin HY, Lansing L, et al (2005). Integrin alphaVbeta3 contains a cell surface receptor site for thyroid hormone that is linked to activation of mitogen-activated protein kinase and induction of angiogenesis. Endocrinology 146:2864–71. 15802494 10.1210/en.2005-0102

[CIT0004] Bonotto M, Gerratana L, Poletto E, et al (2014). Measures of outcome in metastatic breast cancer: insights from a real-world scenario. Oncologist 19:608–15. 24794159 10.1634/theoncologist.2014-0002 PMC4041678

[CIT0005] Danhier F, Le Breton A, Preat V. (2012). RGD-based strategies to target alpha(v) beta(3) integrin in cancer therapy and diagnosis. Mol Pharmaceutics 9:2961–73. 10.1021/mp3002733 22967287

[CIT0006] Du YZ, Cai LL, Liu P, You J, et al. (2012). Tumor cells-specific targeting delivery achieved by A54 peptide functionalized polymeric micelles. Biomaterials 33:8858–67. 22959183 10.1016/j.biomaterials.2012.08.043

[CIT0007] Ducommun B, Lobjois V. (2014). Multicellular tumor spheroid 3D models to decipher cancer cell biology and to evaluate anticancer drugs. Cancer Res 74(19 Suppl):Abstract nr 2025.

[CIT0008] Heerboth S, Housman G, Leary M, et al (2015). EMT and tumor metastasis. Clin Trans Med 4:6. 10.1186/s40169-015-0048-3 PMC4385028 25852822

[CIT0009] Hood JD, Cheresh DA. (2002). Role of integrins in cell invasion and migration. Nat Rev Cancer 2:91–100. 12635172 10.1038/nrc727

[CIT0010] Huang LL, Zhang ZF, Zhang S, Ren JH, et al (2011). Inhibitory action of Celastrol on hypoxia-mediated angiogenesis and metastasis via the HIF-1 alpha pathway. Int J Mol Med 27:407–15. 21249310 10.3892/ijmm.2011.600

[CIT0011] Huang S, DeGuzman A, Bucana CD, Fidler IJ. (2000). Nuclear factor-kappa B activity correlates with growth, angiogenesis, and metastasis of human melanoma cells in nude mice. ClinCancer Res 6:2573–81. 10873114

[CIT0012] Kang CM, Koo HJ, Lee S, et al (2013). 64Cu-Labeled tetraiodothyroacetic acid-conjugated liposomes for PET imaging of tumor angiogenesis. Nucl Med Biol 40:1018–24. 24035550 10.1016/j.nucmedbio.2013.08.003

[CIT0013] Kapoor S. (2016). Tumor growth attenuating effect of Celastrol in systemic malignancies. Int J Cancer 139:1431. 27097353 10.1002/ijc.30151

[CIT0014] Kim DY, Park JW, Ro JY. (2009). Celastrol reduces airway hypersensitivity and lung tissue remodeling via regulating the production of MMP-2/9 in mouse allergic asthma model. Am J Respir Crit Care 179:A4226–27.

[CIT0015] Klein CA. (2009). Parallel progression of primary tumours and metastases. Nat Rev Cancer 9:302–12. 19308069 10.1038/nrc2627

[CIT0016] Liu X, Cheng BL, Meng TT, et al (2016). Synthesis and biological application of BKT-140 peptide modified polymer micelles for treating tumor metastasis with an enhanced cell internalization. Polym Chem 7:1375–86.

[CIT0017] Lu WZ, Jia GF, Zhao C, et al (2015). Celastrol induces apoptosis of HT29 cells through inhibiting miR-21 regulation of Bcl-2 expression. Lat Am J Pharm 34:390–6.

[CIT0018] Matsumoto Y, Sakurai H, Kogashiwa Y, Saito K. (2016). Cetuximab inhibits migration, invasion, metastasis and epithelial-mesenchymal transition but not proliferation via GEP100Arf6-AMAP1 pathway in head and neck squamous cell carcinoma. Cancer Res 76:1605–6.

[CIT0019] Meylan E, Dooley AL, Feldser DM, et al (2009). Requirement for NF-kappa B signalling in a mouse model of lung adenocarcinoma. Nature 462:104–7. 19847165 10.1038/nature08462 PMC2780341

[CIT0020] Mi CL, Shi H, Ma J, et al (2014). Celastrol induces the apoptosis of breast cancer cells and inhibits their invasion via downregulation of MMP-9. Oncol Rep 32:2527–32. 25310109 10.3892/or.2014.3535

[CIT0021] Mo R, Jiang T, DiSanto R, et al (2014). ATP-triggered anticancer drug delivery. Nat Commun 5:3364. 24618921 10.1038/ncomms4364

[CIT0022] Mousa SA, Bergh JJ, Dier E, et al (2008). Tetraiodothyroacetic acid, a small molecule integrin ligand, blocks angiogenesis induced by vascular endothelial growth factor and basic fibroblast growth factor. Angiogenesis 11:183–90. 18080776 10.1007/s10456-007-9088-7

[CIT0023] Nacev A, Kim SH, Rodriguez-Canales J, et al (2011). A dynamic magnetic shift method to increase nanoparticle concentration in cancer metastases: a feasibility study using simulations on autopsy specimens. Int J Nanomedicine 6:2907–23. 22131836 10.2147/IJN.S23724 PMC3224717

[CIT0024] O’Flanagan CH, Rossi EL, McDonell SB, et al (2017). Metabolic reprogramming underlies metastatic potential in an obesity-responsive murine model of metastatic triple negative breast cancer. NPJ Breast Cancer 3:26. 28748213 10.1038/s41523-017-0027-5 PMC5514148

[CIT0025] Pang X, Yi Z, Zhang X, et al (2009). Acetyl-11-keto-beta-boswellic acid inhibits prostate tumor growth by suppressing vascular endothelial growth factor receptor 2-mediated angiogenesis. Cancer Res 69:5893–900. 19567671 10.1158/0008-5472.CAN-09-0755 PMC2724674

[CIT0026] Pang XF, Yi ZF, Zhang J, et al (2010). Celastrol suppresses angiogenesis-mediated tumor growth through inhibition of AKT/mammalian target of rapamycin pathway. Cancer Res 70:1951–9. 20160026 10.1158/0008-5472.CAN-09-3201 PMC2854134

[CIT0027] Park BK, Zhang HL, Zeng QH, et al (2007). NF-kappa B in breast cancer cells promotes osteolytic bone metastasis by inducing osteoclastogenesis via GM-CSF. Nat Med 13:62–9. 17159986 10.1038/nm1519

[CIT0028] Perkins ND. (2007). Integrating cell-signalling pathways with NF-kappa B and IKK function . Nat Rev Mol Cell Biol 8:49–62. 17183360 10.1038/nrm2083

[CIT0029] Rebbaa A, Chu F, Davis FB, et al (2008). Novel function of the thyroid hormone analog tetraiodothyroacetic acid: a cancer chemosensitizing and anti-cancer agent. Angiogenesis 11:269–76. 18386142 10.1007/s10456-008-9110-8

[CIT0030] Sakamoto K, Maeda S. (2010). Targeting NF-kappa B for colorectal cancer. Expert Opin Ther Targets 14:593–601. 20367537 10.1517/14728221003769903

[CIT0031] Schnell O, Krebs B, Wagner E, et al (2008). Expression of integrin alphavbeta3 in gliomas correlates with tumor grade and is not restricted to tumor vasculature. Brain Pathol 18:378–86. 18394009 10.1111/j.1750-3639.2008.00137.x PMC2607528

[CIT0032] Sethi G, Ahn KS, Pandey MK, Aggarwal BB. (2013). Celastrol, a novel triterpene, potentiates TNF-induced apoptosis and suppresses invasion of tumor cells by inhibiting NF-kappa B-regulated gene products and TAK1-mediated NF-kappa B activation. Blood 122:1327. 10.1182/blood-2006-10-050807 17110449

[CIT0033] Sethi G, Sung B, Aggarwal BB. (2008). Nuclear factor-kB activation: from bench to bedside. Exp Biol Med 233:21–31. 10.3181/0707-MR-196 18156302

[CIT0034] Sha M, Ye J, Zhang LX, et al (2014). Celastrol induces apoptosis of gastric cancer cells by miR-21 inhibiting PI3K/Akt-NF-kappa B signaling pathway. Pharmacology 93:39–46. 24434352 10.1159/000357683

[CIT0035] Tan Y, Zhu Zhao Y, Wen Y, et al (2017). Mitochondrial alkaline pH-responsive drug release mediated by Celastrol loaded glycolipid-like micelles for cancer therapy. Biomaterials 154:169–81. 29128845 10.1016/j.biomaterials.2017.07.036

[CIT0036] Ullah I, Muralidharan K, Alkodsi G, et al (2017). Evolutionary analyses of matched primary and metastatic breast cancer reveal both linear and parallel progression with lack of axillary lymph node involvement. Cancer Res 77:P6-01-04.

[CIT0037] Valastyan S, Weinberg RA. (2011). Tumor metastasis: molecular insights and evolving paradigms. Cell 147:275–292. 22000009 10.1016/j.cell.2011.09.024 PMC3261217

[CIT0038] Wang Y, Du H, Zhai G. (2014). Recent advances in active hepatic targeting drug delivery system. Curr Drug Targets 15:573–99. 24606040 10.2174/1389450115666140309232100

[CIT0039] Woodhouse EC, Chuaqui RF, Liotta LA. (1997). General mechanisms of metastasis. Cancer 80:1529–1537. 9362419 10.1002/(sici)1097-0142(19971015)80:8+<1529::aid-cncr2>3.3.co;2-#

[CIT0040] Wu Y, Deng J, Rychahou PG, et al (2009). Stabilization of snail by NF-kappa B is required for inflammation-induced cell migration and invasion. Cancer Cell 15:416–28. 19411070 10.1016/j.ccr.2009.03.016 PMC2881229

[CIT0041] Yang HL, Korivi M, Lin MW, et al (2015). Anti-angiogenic properties of coenzyme Q(0) through downregulation of MMP-9/NF-kappa B and upregulation of HO-1 signaling in TNF-alpha-activated human endothelial cells. Biochem Pharmacol 98:144–156. 26348871 10.1016/j.bcp.2015.09.003

[CIT0042] Zheng L, Fu YY, Zhuang LH, et al (2014). Simultaneous NF-kappa B inhibition and E-cadherin upregulation mediate mutually synergistic anticancer activity of celastrol and SAHA in vitro and in vivo. Int J Cancer 135:1721–1732. 24615207 10.1002/ijc.28810

[CIT0043] Zhou LL, Lin ZX, Fung KP, et al (2011). Celastrol-induced apoptosis in human HaCaT keratinocytes involves the inhibition of NF-kappa B activity. Eur J Pharmacol 670:399–408. 21951963 10.1016/j.ejphar.2011.09.014

[CIT0044] Zhu L, Kate P, Torchilin VP. (2012). Matrix metalloprotease 2-responsive multifunctional liposomal nanocarrier for enhanced tumor targeting. Acs Nano 6:3491–3498. 22409425 10.1021/nn300524f PMC3337349

