# Supplementary material for: Simultaneous targeting therapy for lung metastasis and breast tumor by blocking the NF-κB signaling pathway using Celastrol-loaded micelles
Source: Drug Deliv. 2018 Jan 22;25(1):341–52. doi: 10.1080/10717544.2018.1425778 (PMC6058533; doi:10.1080/10717544.2018.1425778)
Supplement: IDRD_Hu_et_al_Supplemental_Content.docx [file IDRD_A_1425778_SM2132.docx]

**Supporting Information**

**Simultaneous Targeting Therapy for Lung Metastasis and Breast Tumor by Blocking the NF-κB Signaling Pathway Using Celastrol Loaded Micelles.**

Yue Zhao^a^, Yanan Tan^a^, Tingting Meng^b^, Xuan Liu^b^, Yun Zhu^a^, Yun Hong^c^, Xiqin Yang^b^, Hong Yuan^b^, Xuan Huang^d^, and Fuqiang Hu ^a,b,*^

^a^ Ocean College, Zhejiang University, 1 Zheda Road, Zhoushan 316021, China.

^b^ College of Pharmaceutical Science, Zhejiang University, 866 Yuhangtang Road, Hangzhou 310058, China.

^c^ The First Affiliated Hospital, College of Pharmaceutical Medicine, Zhejiang University, Qingchun Road, Hangzhou, China.

^d^ Department of Pharmacy, School of Medicine Science, Jiaxing University, Zhejiang 314001, China.

*** Corresponding Author: Prof. Fuqiang Hu

Tel/Fax: +86-571-88208439

E-mail: [hufq@zju.edu.cn](mailto:hufq@zju.edu.cn)

Table S1

Characteristics of Celastrol-loaded micelles

| Material | Size （nm） | PI | Zeta potential(mV) | EE (%) | DL (%) |  |
| --- | --- | --- | --- | --- | --- | --- |
| CSOSA/Cela | 84.6±1.5 | 0.17±0.03 | 24.3±0.2 | 81.1±7.9 | 14.4±1.9 |  |
| TET-CSOSA/Cela | 82.5±3.6 | 0.06±0.05 | 22.9±0.9 | 73.3±3.9 | 13.7±2.3 |  |

Data represent the mean ± standard deviation (n = 3). PI: polydispersity index.


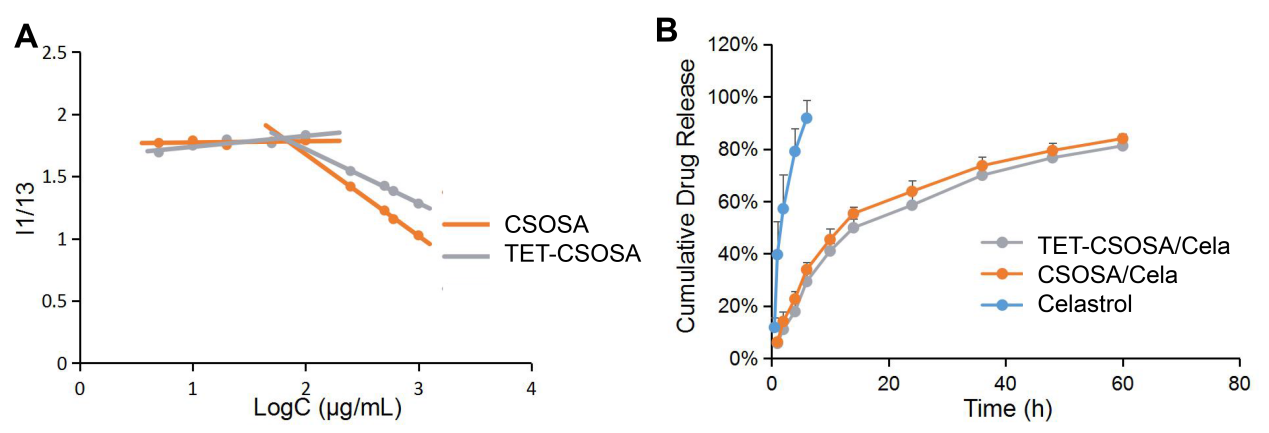


Fig. S1. (A) Characterization of micelles. CMC values of CSOSA and TET-CSOSA micelles. (D) In vitro drug release curve of Celastrol, CSOSA/Cela and TET-CSOSA/Cela, pH=7.4 (n = 3).


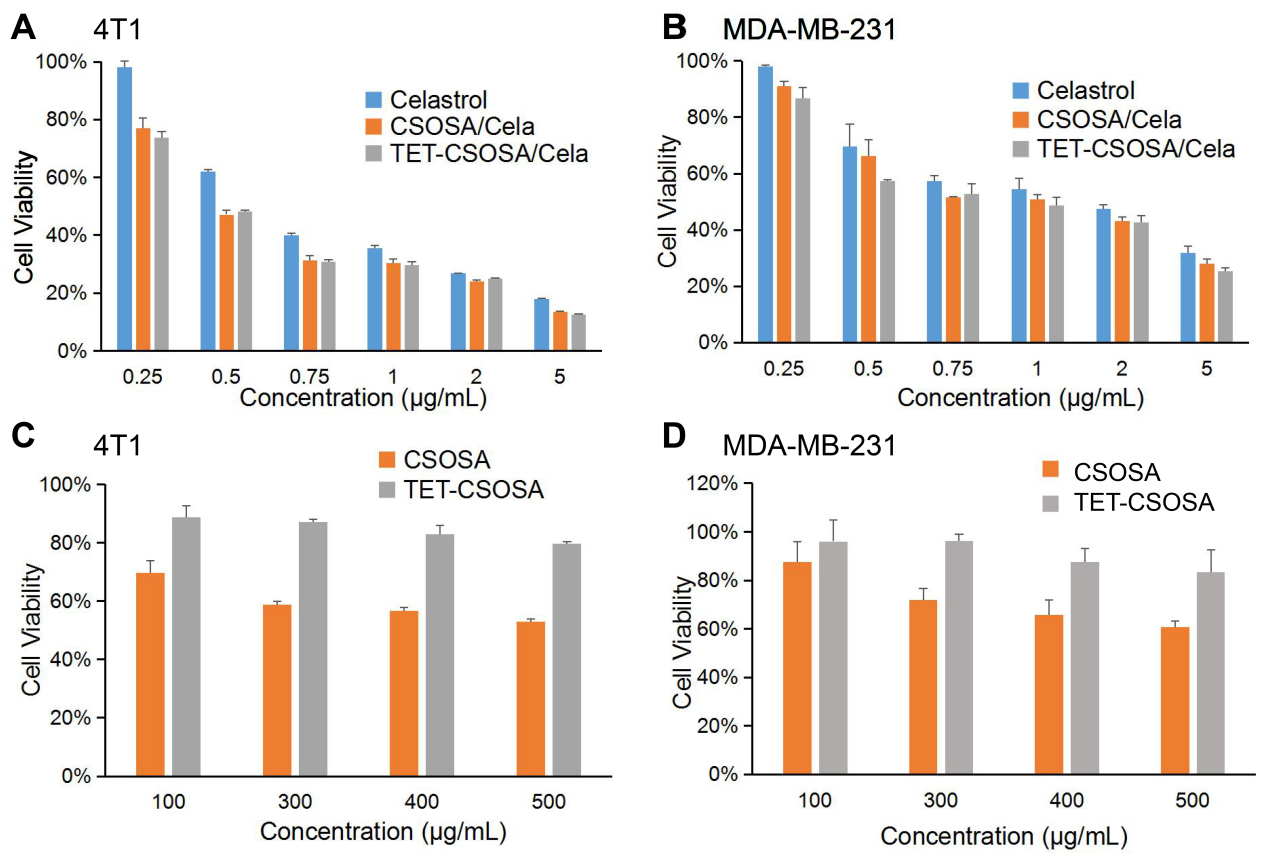


Fig. S2. In vitro cytotoxicity of Celastrol, CSOSA/Cela and TET-CSOSA/Cela against 4T1 (A) and MDA-MB-231 (B) cells for 48 h. Cell viability of different concentrations of CSOSA and TET-CSOSA on 4T1 (C) and MDA-MB-231 (D) cells for 48 h. (n = 3).


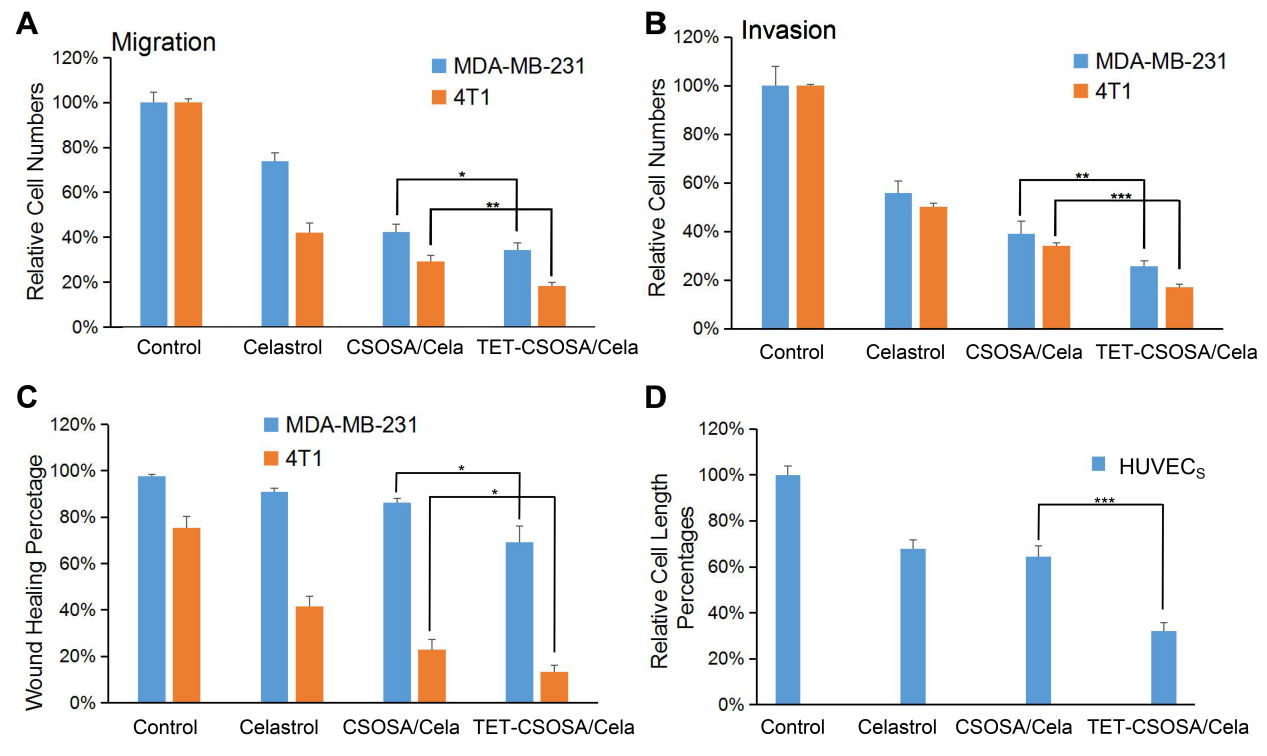


Fig. S3. *In vitro* anti-metastasis effects analysis of different formulations against 4T1 and MDA-MB-231 cells. Relative cell number of migration (A) invasion (B), and wound healing (C) percentage measured by using a microscope. (D) Relative cell length percentage of HUVECs cells in Matrigel with free Cela, CSOSA/Cela and TET-CSOSA/Cela. Wound healing percentage measured by using a microscope. (n = 3, mean ± SD).


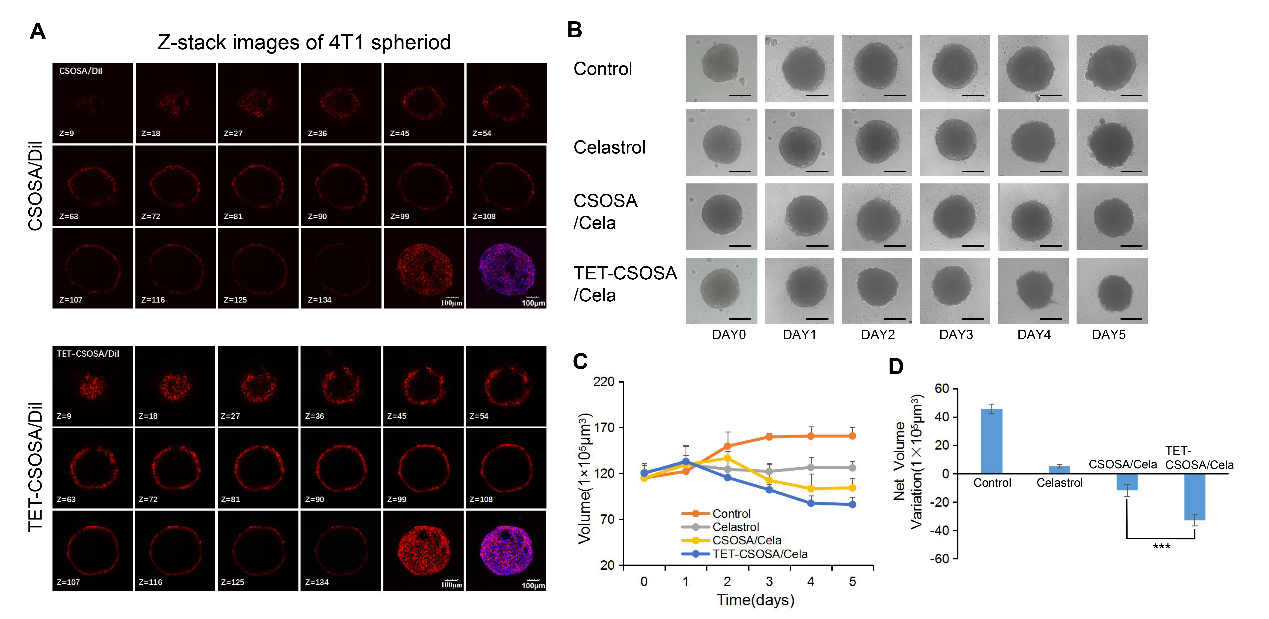


Fig. S4. The inhibition effect of Celasteol-loaded micelles on 4T1 cells spheroids *in vitro*. (A)The representative confocal images were taken after incubation with CSOSA/Dil and TET-CSOSA/Dil micelles. (B) Growth inhibition assay in 4T1 spheroids. Representative images of spheroids observed using an inverted microscope on days 0, 1, 2, 3, 4, and 5 were shown. Scale bar, 200 μm. The volume change line chart (C) and net volume variation (D) between day 5 and day 0 of spheroids were measured after treatment with different groups. (n = 5). Notes: ***, P < 0.001.


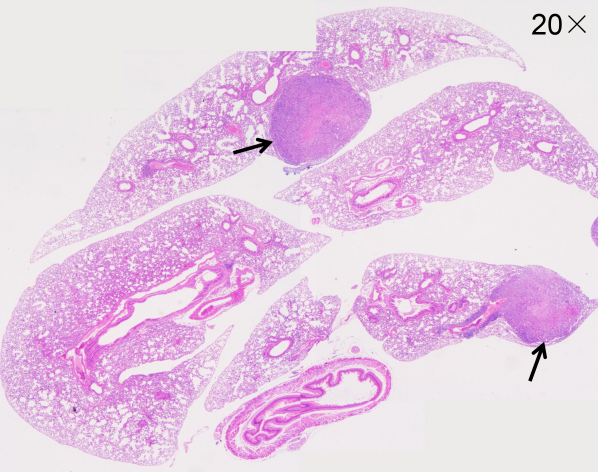


Fig. S5. H&E images of lung tissue when the volume of breast tumor was about 100mm^3^.


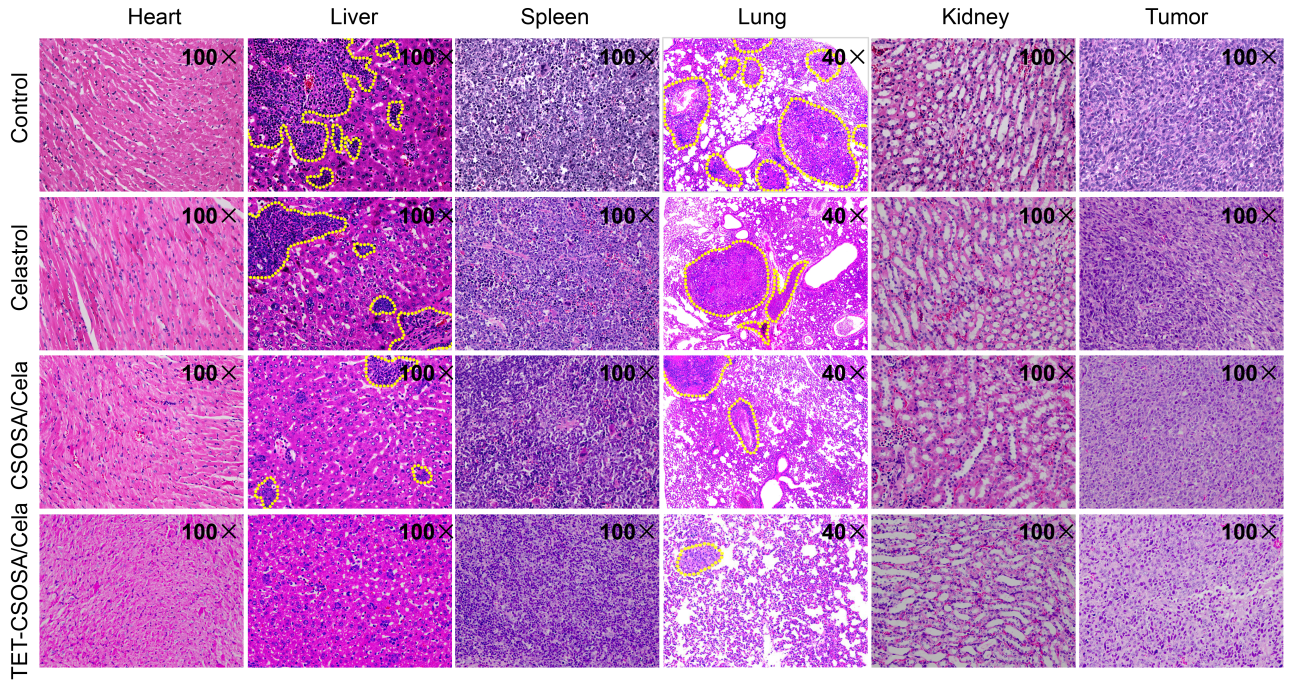


Fig. S6. Representative histological H&E staining of various organ tissues in each treatment group.
